# Steady, Aim, Fire! Optimized Instructions Enhance Performance and Reduce Intra-Trial Variability in a Shooting Task

**DOI:** 10.2478/hukin-2022-000077

**Published:** 2022-11-08

**Authors:** Reza Abdollahipour, William M. Land, Lucia Bizovská, Tomáš Klein, Ludvík Valtr, Miroslav Janura

**Affiliations:** 1 Department of Natural Sciences in Kinanthropology, Faculty of Physical Culture; Palacký University Olomouc, Olomouc, Czech Republic.; 2 Department of Kinesiology, College for Health, Community and Policy, University of Texas at San Antonio, San Antonio, USA.

**Keywords:** external focus, autonomy support, enhanced expectancy, motor performance, shooting

## Abstract

The present study examined the influence of the individual and sequential combination of the key components of OPTIMAL (Optimizing Performance Through Intrinsic Motivation and Attention for Learning) theory (i.e., enhanced expectancies, autonomy support, and external focus), on the performance of a laser-pistol shooting task. In addition to shooting accuracy, intra-trial variability in the sway of forearm/pistol motion prior to movement execution (pulling the trigger) was the primary variable of interest. In a between-within-subject design, thirty-six participants (Mage = 21.27 ± 1.75 years) were randomized into either a control or an optimized group. Enhanced expectancies, autonomy support, and an external focus were implemented via sequential blocks of trials for participants in the optimized group. Participants in the control group performed all trials under “neutral” conditions. Our results showed that motor performance was enhanced for participants in the optimized group compared to those in the control group. Moreover, greater reductions in forearm sway leading up to the trigger pull were observed for the optimized group compared to the control group. These findings suggest higher movement effectiveness and efficiency, potentially through better attunement to task and environmental constraints, when implementing optimized instructions in a self-initiated fine motor task.

## Introduction

Research has shown that OPTIMAL (Optimizing Performance Through Intrinsic Motivation and Attention for Learning) theory for motor learning (Wulf and Lewthwaite, 2016) provides effective guidelines for learners and practitioners who are interested in enhancing performance and learning motor skills. Specifically, OPTIMAL theory proposes that both motivational and attentional factors are central to the enhancement of motor skills. To this extent, these factors are viewed to benefit motor learning and performance through strengthening the coupling between goals and actions. As such, the theory introduces various motivational elements (i.e., enhanced expectancies (EE) and autonomy support (AS)) and attentional components (i.e., external focus (EF) of attention) as the key factors for optimizing motor learning and performance. Enhanced expectancies refer to increases in one’s level of hope, success, or experience, which ultimately produces positive feelings for the performer (Lewthwaite and Wulf, 2010). Autonomy support is suggested to enhance motivation by providing the opportunity for the performer to choose or control the elements of their actions in accordance with their free will (Deci and Ryan, 2008). Both EE and AS are critical for influencing one’s motivation. With respect to attention, an external focus is defined as the performer’s concentration on the external task-relevant information or intended movement effect (object-related information) (Wulf et al., 1998). Independently, each of these motivational and attentional components have been shown to be effective for enhancing performance and learning across various types of motor skills (for a review see Wulf and Lewthwaite, 2016).

Moreover, the sequential combination of any of these two factors such as EE and AS (Wulf et al., 2014), EE and EF (Marchant et al., 2019), or AS and EF (Abdollahipour et al., 2017) have an additive benefit for enhancing both performance and learning of motor skills across a variety of gross motor tasks. Furthermore, recent studies have shown that the sequential combinations of all three factors in successive trial blocks are even more beneficial for motor performance and learning in comparison to combinations of any two factors for gross motor tasks such as ball throwing (Wulf et al., 2018), jumping (Chua et al., 2018), balance (Chua et al., 2020), bowling (Abdollahipour et al., 2020), and golf putting (An et al., 2021) tasks. To date, only few studies have shown beneficial effects of an independent factor such as an EF relative to an internal focus (IF) on enhancing motor performance of fine motor skills (Raisbeck et al., 2020). To our knowledge, no study has examined the individual effects of motivational factors (i.e., AS or EE) or the effects of a sequential combination of EF, AS, and EE (i.e., OPTIMAL theory) on motor performance using a fine motor task. Therefore, investigating the influence of the key components of OPTIMAL theory on a fine motor task would be important as fine motor tasks require a higher degree of precision for controlling and coordination of small muscles (Cuffaro, 2011), which can elicit differential cognitive demands as compared to gross motor control (Raisbeck and Diekfuss, 2015).

While the benefits of OPTIMAL learning are rather robust, the underlying mechanisms of OPTIMAL theory are not fully understood. Recent insights into neuromuscular activity suggest that changes in movement variability and efficiency may underlie benefits to learning and performance. To this extent, research indicates that motivational factors such as AS (Iwatsuki et al., 2021) and an EF (Lohse and Sherwood, 2012) can enhance neuromuscular coordination efficiency, as indicated by reduced muscle activity or electromyography (EMG) signals when producing a comparable amount of force. As such, implementing optimized motivational and attentional factors is associated with increased efficiency of the neuromuscular system. Moreover, such increased efficiency, which is linked to reduced muscle activity, is also likely to reduce unwanted movement variability during task execution. Consequently, reduced movement variability may underlie the benefit associated with OPTIMAL training.

In addition to reductions in movement variability, the movement preparatory period may also be critical for understanding the mechanisms underlying OPTIMAL theory. The movement preparatory period is essential for movement success, in that it involves processing sensory inputs as well as selecting and programming upcoming movement responses (Schmidt et al., 2019). Consequently, one possible path to account for the benefits derived from OPTIMAL theory is to explore the combined effects of attentional and motivational factors on the period prior to movement execution. Providing support for this approach, studies have shown that movement preparation is affected by attentional focus (Ille et al., 2013; Kovacs et al., 2018) and motivational (Meadows et al., 2016) factors independently during reaction time motor tasks, which are externally triggered movements. For example, expert and novice sprinters (Ille et al., 2013) as well as track sprinters (Kovacs et al., 2018) showed reduced pre-motor reaction times (the time interval elapsed from the presentation of the stimulus to the first change in muscle activity) in an external relative to an internal focus condition. In addition, it has been reported that motivation (monetary incentives) reduced premotor reaction time in a handgrip force production task (Meadows et al., 2016). However, despite evidence that suggests differences within the movement preparatory period while manipulating attentional focus and motivational factors during reaction time tasks, it is unclear what changes would occur prior to self-initiated motor tasks, when these factors are implemented in a sequential fashion.

Shedding light on potential changes, neuroimaging evidence has indicated that the onset of activation in the pre-supplementary motor area of the brain before movement execution starts earlier for a self-initiated motor task compared to an externally triggered reaction time motor task (Cunnington et al., 2002). Therefore, it is important to investigate the potential changes in preparation preceding self-initiated motor task, when attentional and motivational factors are implemented as compared to a control condition.

Based on the existing gaps in literature, the purpose of the current study was two-fold. First, the current study examined the effects of individual, and sequential combination of the three key factors of OPTIMAL theory (EE, AS, and EF) (Abdollahipour et al., 2020; Chua et al., 2018, 2020) on motor performance using a fine motor task. The second purpose of the study was to illuminate the underlying mechanisms of OPTIMAL theory by exploring movement variability during the period immediately preceding a self-initiated motor task. To address these aims, participants performed a laser-pistol shooting task under successive blocks emphasizing EE, AS, and EF (optimized instructions) (Abdollahipour et al., 2020; Chua et al., 2018, 2020; Wulf et al., 2018). We hypothesized that the movement outcome (i.e., shooting accuracy) for participants in an optimized instructional group would be better than for participants in a control group (when no optimized instructions are given).

With respect to movement variability prior to task execution, we hypothesized that implementation of optimized instructions would reduce movement variability of forearm/pistol motion prior to movement execution (pulling the trigger) compared to a control group. This hypothesis was based partly on research with rifle shooters that has shown an association between increased shooting accuracy and decreased body sway prior to pulling the trigger (Ball et al., 2003), along with research that has reported improved shooting performance associated with reduced tremor size or movement variability in the pistol and distal arm planes (Lakie, 2010; Mononen et al., 2007; Tang et al., 2008).

## Methods

### Participants

Thirty-six healthy undergraduate university students (20 males and 16 females, age range 19–27 years) with a mean age of 21.27 ± 1.75 years and a mean height of 174.05 ± 8.60 cm participated in the study. Participants did not have any previous experience with the task. An a priori power analysis with G*Power 3.1 indicated that 36 participants would be sufficient to identify significant differences in the dependent variables in a two-factor mixed-design analysis of variance (ANOVA) with a power (1 - β) of .90, effect size ƒ of .25 (η_ρ_^2^ = .06), and an α level of .05 (Faul et al., 2007). Ethical approval was obtained from the university’s internal review board prior to conducting the experiment. Written informed consent was obtained from all participants prior to data collection. Participants were provided general information about the experiment, but were not aware of the specific purposes of the study.

### Apparatus and Task

The task consisted of shooting with a laser pistol (Apeom laser pistol, APL 160, APEOM s. r. o., Ostrava, Czechia) at a target as accurately as possible. Participants were asked to hold the laser-pistol with their dominant hand. The hand-dominancy was self-indicated by the participant. Next, they were required to stand behind a starting line, which was placed 5 m away from the target, point the pistol at the target, and then pull the trigger.

An optoelectronic target (Target LPT, APEOM s. r. o., Ostrava, Czechia) was mounted on a tripod at the eye level of each participant. The dimensions of the target were 20 cm height x 20 cm width x 22 cm depth. The target had a sensor to detect the location of the optical shots from the laser pistol, and then evaluated the position and the score of shots using the supplied display. The target was connected to a laptop via a Bluetooth system, and the score of each shot was shown on a laptop display. Participants were able to briefly see the location of each shot via laser illumination on the target. However, participants were not able to see their scores displayed on the laptop after each shot. The target consisted of 10 concentric circles with a bull’s eye in the center. The first central circle had a 0.55 cm radius surrounded by concentric circles with radii of 1.35, 2.15, 2.95, 3.75, 4.55, 5.35, 6.15, 6.95, 7.75 cm, respectively. Each shot was scored based on a point system depending on the location within each ring the shot landed. Scores for shots that landed within the bull’s eye could range from 10 to 10.9 in 0.1 increments depending on how close the shot came to the center of the target (larger values represent more accurate shots). For the next concentric ring, scores could range from 9 to 9.9 depending on how close the shot came from reaching the next inner circle. Ranges in scores continued (8–8.9, 7–7.9, 6–6.9, etc) for each subsequent ring on the target. Any shot that landed outside the last concentric circle on the target was scored as 0.

An eight-camera Vicon Vantage V5 motion capture system (Oxford Metrics, UK) was used to collect kinematic data at 200 Hz. To investigate the movement variability of the forearm and the pistol, one retroreflective marker was placed directly on the skin of the participant's forearm on the posterior surface of the forearm midpoint between the wrist joint center and the elbow joint center, as movement variability at the forearm could be compensatory for stabilizing the movement at the pistol (Arutyunyan et al., 1968). The second marker was attached to the bottom side of the pistol barrel. The XYZ coordinates (i.e., anterior-posterior, media-lateral, and vertical planes) of these markers were used to determine the position of the forearm and the laser pistol. The laser pistol and the Vicon kinematic system were synchronized in order for Vicon to detect the moment of the trigger pull for each shot.

### Procedure

First, the retroreflective markers were attached to the forearm and the bottom side of the pistol barrel. Next, the experimenter provided basic descriptive instructions of the task. Specifically, the participant was provided with the following instructions: a) stand behind the starting line, b) hold the laser pistol at one’s side with the dominant hand, c) raise and point the pistol towards the target and then pull the trigger. Subsequently, the experimenter demonstrated the task to the participant. Participants were asked to shoot as accurately as possible with the laser pistol at the center of the target. Next, each participant performed three familiarization shots sequentially. Participants kept the pistol raised towards the target between each shot, and did not lower the pistol to their side between shots. Following the familiarization trials, they were then asked to perform ten shots sequentially during a baseline condition. The time between each shot was self-paced. The experimenter monitored the number of shots and scores for each shot registered on the laptop. This procedure enabled us to detect the shots that missed the target as well as the correct number of shots in each block.

Participants were pseudo-randomly divided (i.e., equally, and evenly matched based on gender) into two groups: the optimized group and the control group. The gender-matched assignment was performed to eliminate any potential concern in shooting performance accuracy between males and females (Mon-López et al., 2020). All participants performed 180 shots in total during an intervention phase in which they were given specific instructional sets. For the optimized group, participants performed the task across three instructional conditions (EE, AS, and EF) with each condition consisting of sixty shots divided into six blocks of 10 shots (60 shots per condition x three conditions). Each of the three conditions had their own set of instructions. Particularly, in the EE condition, participants were told, “you are doing very well”. The frequency of EE was given before the beginning of each block. In the EF condition, participants were asked to “focus on the bull’s eye” before each block. In the AS condition, participants were informed that they could choose the number of shots in each block (12, 11, 10, 10, 9, and 8), indicating that they could make a choice before each trial in the block. These numbers were written on individual cards and each participant showed one of these cards before each block. Then, the participant performed the chosen number of shots in that block. For the next block, participants should choose the number of the shots from the remaining cards (total number of shots always equaled 60). The order of all three experimental conditions for the optimized group including EE, AS, and EF was counterbalanced across participants.

Participants from the control group performed all blocks without receiving any specific motivational or attentional focus instructions. The only instructions participants received pertained to a basic description of the task procedures and goals as described above. To control for the varying number of shots in each block for the AS condition, each control group participant was yoked to a participant in the optimized group. That is, before each trial participants were asked to perform the same number of shots as his/her respective counterpart had chosen during the AS condition in the optimized group. There was a 15-s rest interval between blocks, and a 3-min rest interval between each condition (after a block of 60 shots in the control group) for both groups.

### Data analysis

Shooting accuracy was the primary variable of interest for assessing the outcome performance. To assess initial performance levels of the two groups (control and optimized) at baseline, the score for each participant was averaged across all ten-baseline trials. To compare the influence of optimized instructions on shooting accuracy during the intervention phase, the score for each participant was averaged across all three optimized conditions (e.g., one-hundred-eighty trials). Preliminary Shapiro-Wilk tests showed that the assumption of normality was violated (*p* < .05). Accordingly, a non-parametric Mann-Whitney *U* test was used to examine group differences in mean ranks (*M*Rank) between the groups. To estimate the effect sizes between optimized and control groups, the *r* effect size was calculated by dividing the *z’s* score value by the square root of the sample size number (*N* = 36) (Fritz et al., 2012). For consistency in reporting effect sizes, *r* values were transformed to Cohen’s *d* values (Cohen, 1988; Lenhard and Lenhard, 2016).

To analyse the unique effects of each condition (AS, EE, and EF) on motor performance within the optimized group, the average score of each condition was calculated for each participant. Shapiro-Wilk tests indicated that the assumption of normality was violated (*p* < .05). Therefore, a Friedman and Dunn-Bonferroni post hoc test was used to evaluate the differences in mean ranks (*M*Rank) among the three conditions. The effect sizes for the non-parametric Friedman test were estimated using Kendall’s *W* (or Kendall’s coefficient of concordance), with values ranging from 0 (no relationship) to 1 (a perfect relationship) and higher (a strong relationship) (Field, 2009).

To assess intra-trial variability of forearm and pistol sway prior to each shot, the degree of spatial movement variability was calculated during four time periods leading up to the trigger pull (750-560 ms, 560-370 ms, 370-180 ms, and 180-0 ms before the shot). The maximum of 750 ms before the shot was chosen for computation as this time interval was in compliance with the minimum time between shots performed by all participants. For each time period, standard deviations were calculated for the spatial position of each marker (forearm and pistol) in each of the three coordinate directions (anterior-posterior, media-lateral, and vertical), separately. Preliminary Shapiro-Wilk tests showed that data were distributed normally (*p* > .05). Subsequently, the standard deviations for each marker (forearm and pistol), and for each direction, were analysed separately using a two (groups: optimized vs. control) x four (time periods) mixed-model ANOVA with repeated measures on the last factor.

To assess the unique impact of each condition (AS, EE, and EF) on movement variability within the optimized group only, a three (condition: EF, AS, and EE) x four (time period: 750-560 ms, 560-370 ms, 370-180 ms, and 180-0 ms) repeated measures (RM) ANOVA was performed for each movement direction separately. Shapiro-Wilk tests showed that data were distributed normally (*p* > .05).

Bonferroni adjustments and pairwise comparison post-hoc tests were used for ANOVA, when appropriate. The Mauchly’s test was used for assessing the violation of the assumption of sphericity. Therefore, Greenhouse-Geisser values were applied, when the Mauchly’s test was violated (*p* > .05). Effect size values for ANOVA were quantified using partial eta squared (η_ρ_2), where η_ρ_^2^ = .01, .06, and .14 corresponded to a small, moderate, and large effect, respectively (Cohen, 1988). To estimate the effect sizes between time periods, the repeated-measures version of Cohen’s *d* was utilized. Particularly, Cohen’s *d* was utilized as a measure of the difference between group means using the repeated-measures version of Cohen’s *d* that factors in the correlation between time periods (Morris and DeShon, 2002). The evaluation of Cohen’s *d* corresponded to a low (*d* = 0.2), medium (*d* = 0.5), and large (*d* = 0.8) effect (Cohen, 1988). The level of significance for all statistical analyses was set at α = .05. All analyses were performed using IBM SPSS software (version 21.0; IBM, Armonk, NY). Finally, three participants were excluded from kinematic analysis due to missing data. As such, a total of thirty-three participants were included in the kinematic analyses.

## Results

### Outcome performance

**Figure 1:**
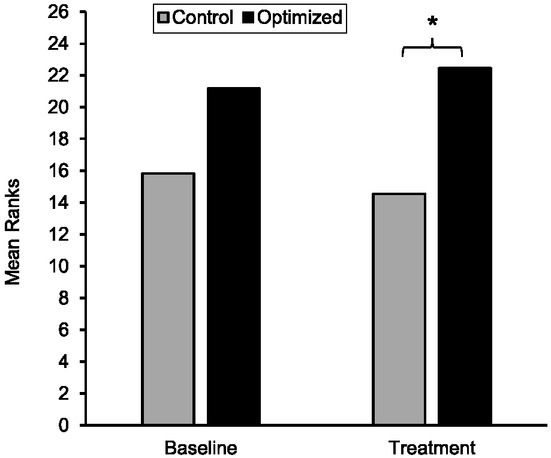
Mean ranks of accuracy scores of shots for the optimal and control groups during baseline and performance. Note: Higher mean ranks indicate better accuracy scores.

The results of the Mann-Whitney *U* test at baseline showed that there were no significant differences in shot accuracy between the optimized (*M*Rank = 21.17) and control (*M*Rank = 15.83) groups, (*U* = 210.00, *z* = 1.519, *p* = .134, *d* = 0.523). The results of an independent t-test showed that there was no significant difference between participants in optimized (M = 7.39 ± 1.45) and control (M = 6.33 ± 2.13) groups at pretest (t = -1.742, *p* = .091, *d* = 0.581 (see Figure 1).

With respect to the intervention phase, the results of the Mann-Whitney *U* test indicated a significant difference between the two groups (*U* = 233.0, *z* = 2.246, *p* = .024, *d* = 0.806), indicating higher accuracy scores for the optimized group (*M*Rank = 22.44) relative to the control group (*M*Rank = 14.56) The results of an independent t-test showed that there was a significant difference between participants in optimzed (M = 8.32 ± 0.72) and control (M = 7.72 ± 0.83) groups at the intervention phase (t = -2.271, *p* = .030, *d* = 0.772).

The results of the Friedman test on the unique impact of each of the three instructional conditions including EE (*M*Rank = 2.06), AS (*M*Rank = 1.89), and EF (*M*Rank = 2.06) on shot accuracy within the optimized group did not show a significant difference between the conditions, *χ2* (2, *N* = 18) = 0.333, *p* = .846, Kendall’s *W* = .009. As such, each condition equally contributed to the overall improvement in shooting accuracy with respect to the control group.

### Movement variability

#### Forearm

The main effect of group, time periods, and interactions of groups and time periods for movement variability of the forearm in the anterior-posterior, medial-lateral and vertical directions at baseline failed to reach significance (all *p* > .05).

**Figure 2:**
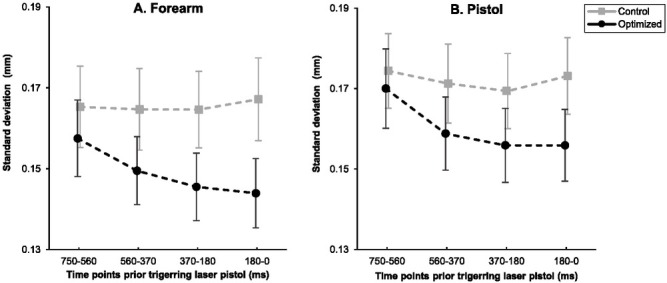
Mean standard deviation of the forearm (A) and the pistol (B) position in the anterior posterior axis for the optimized and control groups across time periods (750–560 ms, 560–370 ms, 370–180 ms, and 180–0 ms) prior to the trigger pull during the intervention phase. Note: Error bars represent standard error.

Figure 2A illustrates the movement variability of the forearm between optimized versus control groups during the intervention phase in the anterior posterior direction leading up to the trigger pull. Results indicated that the main effect of groups, *F*(1, 31) = 1.213, *p* = .279, η_ρ_^2^ = .038, and time periods, *F*(1.974, 61.189) = 2.598, *p* = .083, η_ρ_^2^ = .077, were not significant for the movement variability of the forearm in the anterior posterior axis. However, the interaction between the group and time periods was significant, *F*(1.974, 61.189) = 3.217, *p* = .048, η_ρ_^2^ = .094. As such, there was a significant reduction in movement variability of the forearm in the anterior posterior direction for the optimized group leading up to the trigger pull, *F*(1, 16) = 16.873, *p* = .001, η_ρ_^2^ = .513 (Figure 2A). In contrast, forearm variability was relatively unchanged in the anterior posterior direction leading up to the trigger pull for the control group, *F*(1, 15) = .090, *p* = .768, η_ρ_^2^ = .006.

**Figure 3:**
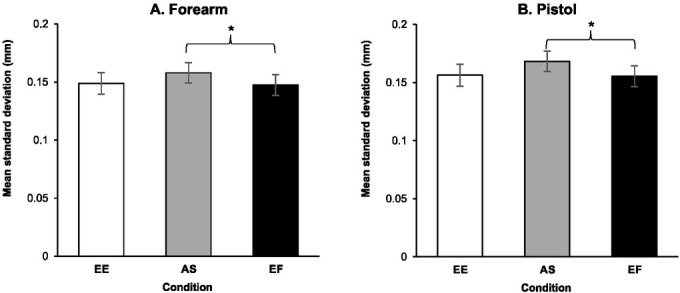
Movement variability of the forearm (A) and the pistol (B) during the intervention phase for different conditions within the optimized group: enhanced expectancy (EE), autonomy support (AS), and external focus (EF). Error bars represent standard errors.

Results from the RM ANOVA on the unique contributions of each condition to forearm sway variability in the anterior posterior plane for the optimized group indicated that the main effect of condition was significant, *F*(2, 32) = 3.492, *p* = .042, η_ρ_^2^ = .179. Post-hoc analysis indicated that variability during the EF condition (*M* = 0.148 ± 0.036 mm) was significantly less than during the AS condition (*M* = 0.158 ± 0.036 mm, *p* = .044, *d* = 0.637). No significant differences were found between movement variability during the EF condition relative to the EE condition (*M* = 0.149 ± 0.038 mm, *p* > .99, *d* = 0.046) and between AS and EE conditions (*p* = .083, *d* = 0.551) (Figure 3A). Also, the main effect of time periods was significant, *F*(3, 48) = 8.666, *p* < .001, η_ρ_^2^ = .351. As observed in Figure 3A, there was a significant linear decline in movement variability in the anterior posterior direction across time leading up to the trigger pull, *F*(1, 16) = 16.679, *p* = .001, η_ρ_^2^ = .510. Finally, the interaction between conditions and time periods failed to reach significance, *F*(3.047, 48.753) = .950, *p* = .425, η_ρ_^2^ = .056.

#### The pistol

The main effect of the group, time periods, and interactions of groups and time periods for movement variability of the pistol in the anterior-posterior, medial-lateral and vertical directions at baseline failed to reach significance (all *p* > .05).

During the intervention phase, the main effect of the group, *F*(1, 31) = 0.830, *p* = .369, η_ρ_^2^ = .026, and the interactions between groups and time periods *F*(2.191, 67.911) = 2.191, *p* = .115, η_ρ_^2^ = .066, failed to reach significance in the anterior posterior axis for the pistol. However, the main effect of time periods in the anterior posterior direction for the pistol was significant, *F*(2.191, 67.911) = 5.026, *p* =.008, η_ρ_^2^ = .140. More specifically, there was a significant linear decline in the movement variability of the pistol in the posterior anterior direction leading up to the trigger pull, *F*(1, 31) = 5.074, *p* = .032, η_ρ_^2^ = .141 (Figure 2B). That is, participants steadied the pistol right before the trigger pull.

Results from the RM ANOVA on the unique contributions of each condition to pistol sway variability in the anterior posterior plane for the optimized group indicated that the main effect of condition was significant, *F*(2, 32) = 4.520, *p* = .019, η_ρ_^2^ = .220. Post-hoc analysis indicated that the variability during the EF condition (*M* = 0.155 ± 0.037 mm) was significantly less than during the AS condition (*M* = 0.168 ± 0.041, *p* = .007, *d* = 0.855). No significant differences were found between movement variability during the EF condition relative to the EE condition (*M* = 0.156 ± 0.037 mm, *p* > .99, *d* = 0.047) and between AS and EE conditions (*p* = .054, *d* = .685) (Figure 3B). Also, the main effect of time periods was significant, *F*(3, 48) = 11.690, *p* < .001, η_ρ_^2^ = .442. More specifically, as observed in Figure 2B, there was a linear decline in movement variability in the anterior posterior direction of pistol motion leading up to the trigger pull, *F*(1, 16) = 21.388, *p* < .001, η_ρ_^2^ = .572. No significant differences were found for the interactions of condition and time periods in the movement variability of the pistol in the anterior posterior direction across the time leading up to the trigger pull, *F*(6, 96) = 0.493, *p* = .813, η_ρ_^2^ = .030.

During the intervention phase, the main effect of the group, time periods, and interactions of groups and time periods in the movement variability of the pistol or the forearm in the medial-lateral and vertical directions leading up to the trigger pull were not significant (all *p* > .05). Also, no significant difference was found for the unique impact of each of the three instructional conditions on shot accuracy within the optimized group in the medial-lateral and vertical directions in the movement variability of the pistol or the forearm leading up to the trigger pull during the intervention phase (all *p* >.05).

## Discussion

The primary aim of the current study was to examine the effect of sequential combination (via sequential blocks) of three key components of OPTIMAL theory (Wulf and Lewthwaite, 2016) on motor performance in a fine-motor task. Our results support the findings of previous research on gross motor skills (Abdollahipour et al., 2020; Chua et al., 2018, 2020) which show that motor performance is enhanced when these three factors are implemented in successive trial blocks for an optimized group in comparison to a control group. Stemming from this, our findings indicate that regardless of the differences in cognitive demands between fine and gross motor skills (Raisbeck and Diekfuss, 2015), implementing the components of OPTIMAL theory has benefits for motor performance across a variety of task types. The present findings are also in line with the results of previous studies which have shown performance under a control condition to be insufficient to reach an optimal performance outcome when compared to a sequential combination of two or three factors of OPTIMAL theory (e.g., Abdollahipour et al., 2020; Chua, et al., 2018; Marchant et al., 2019; Wulf et al., 2018). That is, as human performance is a blend of social-cognitive-affective-motor factors (Lewthwaite and Wulf, 2010), certain combinations of psychological factors such as motivation (e.g., EE and AS) and attention (e.g., EF) should be provided to enable individuals to reach an optimized performance outcome (Wulf and Lewthwaite, 2016).

The second aim of the study was to investigate the effects of optimized instructions on the variability in the sway of forearm/pistol motion prior to movement execution (pulling the trigger). Our results indicated that intra-trial movement variability of the forearm in the anterior-posterior direction reduced over time leading up to the trigger pull for the optimized group. In contrast, no such reductions in movement variability were observed for participants in the control group. This is an important finding as even minor displacement of the limbs or the body could result in degraded shot accuracy (Lakie, 2010; Mononen et al., 2007; Tang et al., 2008). In fact, reduced variability of forearm movements leading up to the trigger pull may be reflective of better attunement to environmental and task constraints, thus facilitating goal-action coupling or task-focus. This reduction in variability is consistent with previous research that reported increased shooting accuracy corresponding with reductions in intra-trial variability in the body sway of rifle shooters (Ball et al., 2003). In addition, reduction in intra-trial variability of forearm motion during the preparatory period in the optimized group corroborates the results of previous studies on the influence of attentional (Ille et al., 2013; Kovacs et al., 2018) and motivational (Meadows et al., 2016) factors on movement preparation in externally triggered reaction time tasks.

Although the results of the current study reveal reductions in movement variability during the preparatory phase, we are unable to precisely distinguish the point at which the pre-motor phase (period before the first change in muscle activity) ends and the beginning of the motor phase (period after movement initiation). That is, there would be a small amount of initial finger movement acting on the trigger before the shot was fired, thus ending the trial. To precisely determine the initiation of the motor phase, the use of EMG or functional magnetic resonance imaging measures would be required. Being able to draw a distinction between the pre-motor and motor phase would help determine what potential changes occur in brain regions such as the supplementary motor area or the pre-motor cortex (Cunnington et al., 2002) when implementing the OPTIMAL theory components. Despite not being able to distinguish between the pre-motor and the initiation of the motor phase in the current study, we assume that the main reduction in movement variability in the optimized group likely started during the pre-motor phase (prior to initiation of the trigger pull with the index finger), as the reductions in variability were already observable between the periods 750–560 ms and 560–370 ms prior to the trigger pull. Overall, our findings suggest that the key components of OPTIMAL theory may substantially influence movement preparation (most probably the pre-motor phase) in a self-initiated motor task, which in turn influences forthcoming performance. More specifically, during the movement preparation period, OPTIMAL theory components may be acting to facilitate attunement of the motor system to continuous perceptual information and environmental and task constraints thus resulting in reduced movement variability leading up to the trigger pull (Davids et al., 2008; Land et al., 2013).

In the current research, there was no difference in intra-trial movement variability in the medial-lateral and vertical directions for either the forearm or the pistol. This result is not surprising as differences in the media-lateral direction of pistol motion may not always be a critical factor for aiming a pistol at the target. For example, research by Ko et al. (2018) examined the variability of body posture and pistol motion in novice and skilled rifle shooters, and found no differences in the standard deviation of pistol motion within the media-lateral direction for aiming the pistol at the target. However, the researchers did find differences in the variability of body posture and pistol motion in the anterior-posterior direction, consistent with the findings of the present study. Thus, it appears that there could be a critical compensatory adjustment in the anterior-posterior direction, which primarily accounts for the difference between good and poor performance in a pistol-aiming task.

With regard to the individual effects of AS, EE, and EF conditions on the performance of the optimized group, there was no difference between the conditions on shot accuracy. However, there was a significant difference between the conditions in the movement variability of the forearm and the pistol. Specifically, the results indicated reduced variability during the EF relative to AS conditions. This finding suggests that EF instructions contributed more to the overall reduction in movement variability relative to AS. However, it should be noted that the differences in movement variability between these two conditions did not translate to significant differences in shooting accuracy between the two. Reductions in movement variability in and of itself do not always directly translate into improved accuracy scores, but rather is a component contributing to overall performance levels. More importantly, however, examination of movement variability can provide insight into the mechanisms underlying OPTIMAL theory’s influence on motor functioning.

The findings of the current study support existing research indicating that a sequential combination of three variables of OPTIMAL theory in successive blocks promotes motor performance in both fine and gross motor skills (Abdollahipour et al., 2020; Chua et al., 2018, 2020). However, a gap in knowledge still exists on the extent to which these variables are effective when combined concurrently. Therefore, we recommend that future research should examine the combination of all three variables simultaneously in one set of instructions on performance and learning of motor skills.

## Conclusion

Overall, the findings of the current study indicate that implementing the three components of OPTIMAL theory (EE, AS, and EF) together in successive trial blocks improves motor performance in self-initiated fine motor tasks. Moreover, the result of the movement variability analysis showed reductions in intra-trial variability of forearm motion leading up to the trigger pull for the optimized learning group. This finding suggests higher movement efficiency, potentially through better attunement to task and environmental constraints, when implementing optimized instructions compared to a control group. However, future research is needed to substantiate the influence of optimized instructions on movement efficiency and constraint attunement. To this extent, future research utilizing muscle activity and brain neuroimaging, or brain stimulation techniques may be beneficial for illuminating the neuromuscular and cognitive/neural mechanisms underlying the benefits of instructions based on OPTIMAL theory factors.
